# Polymorphisms in *Anopheles gambiae* Immune Genes Associated with Natural Resistance to *Plasmodium falciparum*

**DOI:** 10.1371/journal.ppat.1001112

**Published:** 2010-09-16

**Authors:** Caroline Harris, Louis Lambrechts, François Rousset, Luc Abate, Sandrine E. Nsango, Didier Fontenille, Isabelle Morlais, Anna Cohuet

**Affiliations:** 1 Characterization and Control of Vector Populations, Institut de Recherche pour le Développement, Montpellier, France; 2 Department of Virology, Institut Pasteur, Paris, France; 3 Institut des Sciences de l'Évolution, Université Montpellier 2, CNRS, Montpellier, France; 4 Laboratoire de Recherche sur le Paludisme, Institut de Recherche pour le Développement, IRD-OCEAC, Yaoundé, Cameroun; 5 Institut de Recherche en Sciences de la Santé, Bobo Dioulasso, Burkina Faso; Stanford University, United States of America

## Abstract

Many genes involved in the immune response of *Anopheles gambiae*, the main malaria vector in Africa, have been identified, but whether naturally occurring polymorphisms in these genes underlie variation in resistance to the human malaria parasite, *Plasmodium falciparum*, is currently unknown. Here we carried out a candidate gene association study to identify single nucleotide polymorphisms (SNPs) associated with natural resistance to *P. falciparum*. *A. gambiae* M form mosquitoes from Cameroon were experimentally challenged with three local wild *P. falciparum* isolates. Statistical associations were assessed between 157 SNPs selected from a set of 67 *A. gambiae* immune-related genes and the level of infection. Isolate-specific associations were accounted for by including the effect of the isolate in the analysis. Five SNPs were significantly associated to the infection phenotype, located within or upstream of *AgMDL1, CEC1, Sp PPO activate, Sp SNAKElike*, and *TOLL6*. Low overall and local linkage disequilibrium indicated high specificity in the loci found. Association between infection phenotype and two SNPs was isolate-specific, providing the first evidence of vector genotype by parasite isolate interactions at the molecular level. Four SNPs were associated to either oocyst presence or load, indicating that the genetic basis of infection prevalence and intensity may differ. The validity of the approach was verified by confirming the functional role of *Sp SNAKElike* in gene silencing assays. These results strongly support the role of genetic variation within or near these five *A. gambiae* immune genes, in concert with other genes, in natural resistance to *P. falciparum*. They emphasize the need to distinguish between infection prevalence and intensity and to account for the genetic specificity of vector-parasite interactions in dissecting the genetic basis of *Anopheles* resistance to human malaria.

## Author Summary

*Anopheles gambiae* is the main malaria vector in Africa, transmitting the parasite when it blood feeds on human hosts. The parasite undergoes several developmental stages in the mosquito to complete its life cycle, during which time it is confronted by the mosquito's immune system. The resistance of mosquitoes to malaria infection is highly variable in wild populations and is known to be under strong genetic control, but to date the specific genes responsible for this variation remain to be identified. The present study uncovers variations in *A. gambiae* immune genes that are associated with natural resistance to *Plasmodium falciparum*, the deadliest human malaria parasite. The association of some mosquito genetic loci with the level of infection depended on the *P. falciparum* isolate, suggesting that resistance is determined by interactions between the genome of the mosquito and that of the parasite. This finding highlights the need to account for the natural genetic diversity of malaria parasites in future research on vector-parasite interactions. The loci uncovered in this study are potential targets for developing novel malaria control strategies based on natural mosquito resistance mechanisms.

## Introduction

Human malaria is transmitted by female *Anopheles* mosquitoes, which vary in vector competence at both the species and individual level [1]. In *Anopheles gambiae*, the main malaria vector in Africa, it has been possible to select laboratory strains for their resistance or susceptibility to *Plasmodium* infection [2], [3], indicating that resistance has a genetic basis. This led to much effort being targeted towards understanding the genetic determinants of resistance with the hope of uncovering novel ways to reduce malaria transmission [4]. Although considerable progress has been made in model systems, the genetic basis of *Anopheles* resistance to *Plasmodium* remains to be understood in detail in epidemiologically meaningful vector-parasite species combinations. Resistance of natural populations of *A. gambiae* to *Plasmodium falciparum*, the deadliest human malaria parasite, is currently under scrutiny.

The development of powerful genetic tools [5]–[7] in parallel with the sequencing of the *A. gambiae* genome [8] has substantially improved our knowledge of the molecular interactions between *Anopheles* and *Plasmodium*. It was shown that mosquito innate immunity plays a major role in controlling the level of infection by eliminating the majority of malaria parasites (reviewed in [9]). The general scheme of the *A. gambiae* immune response has been deciphered: initially, pattern recognition receptors (PRRs) bind to pathogen-associated molecular patterns of the parasite that trigger signal transduction and modulation cascades; finally, effector molecules are activated to kill the parasites through a range of possible mechanisms [10]. The outcome of infection seems to depend on a fine balance between mosquito factors that act either positively or negatively on *Plasmodium* development [11]–[21].

Phenotypic variation in *A. gambiae* resistance to *P. falciparum* is likely influenced by naturally occurring polymorphism in genes that encode positive or negative modulators of the immune response. For instance, genetic variation at pathogen recognition and intracellular signaling loci may significantly contribute to phenotypic variation in immune competence [22]. If some mosquito immune variants are expected to perform better in controlling malaria infection, they are however not expected to reach fixation for at least two reasons. Firstly, even if not clearly documented in the *A. gambiae - P. falciparum* couple, the mosquito immune response is likely to be costly [23], [24], which may counteract the selection pressure exerted by the parasite and maintain the frequency of resistance at intermediate levels [25]. Secondly, interactions between *A. gambiae* and *P. falciparum* appear to be genotype-specific. Experiments using different *A. gambiae* families challenged with several field isolates of *P. falciparum* revealed significant mosquito genotype by parasite genotype (G x G) interactions, whereby the outcome of infection depends on the specific combination of mosquito and parasite genotypes [26]. Such G x G interactions can promote the maintenance of polymorphism through negative frequency-dependant selection [27].

Earlier studies exploring the genetic variation underlying *Anopheles* resistance to *Plasmodium* have mainly relied on Quantitative Trait Loci (QTL) mapping strategies. This has generally been conducted in model systems by exposing selected resistant/susceptible *A. gambiae* strains to rodent or simian *Plasmodium* species [28]–[30]. The most recent study identified loci associated to resistance in the chromosomal region containing *TEP1* [6], a gene that was previously shown to play a major role in the mosquito immune response [11], [13], [31] and in *P. falciparum* development [14]. However, mechanisms of anti-*Plasmodium* defense in mosquitoes initially uncovered in model systems do not always hold for the natural couple *A. gambiae* - *P. falciparum* [32], [33], likely because of the absence of a shared evolutionary history in artificial species combinations [34], [35].

Studies of the natural *A. gambiae* - *P. falciparum* couple are still limited in number but have identified promising genetic markers of resistance. Associations were found between *NOS* and *cecropin* gene polymorphisms and infection status in wild mosquitoes from East Africa [36], while a QTL mapping approach identified a *Plasmodium* Resistance Island (PRI) on chromosome 2L in mosquitoes from both East and West Africa [37], [38]. Fine genetic dissection within the PRI highlighted a family of three genes (*APL1A*, *APL1B* and *APL1C*) with different roles in infection control. APL1A affects *P. falciparum* development and APL1C limits the development of the rodent parasite *Plasmodium berghei* [15], [39] and is part of the important LRIM1/APL1C/TEP1 complex that is known to play a central role in controlling *Plasmodium* infection in *A. gambiae* [11], [31]. More stringent tests have shown polymorphic sites within APL1C to account for much of the variation in immunity against *P. berghei* [39].

To date, studies on the natural resistance of *A. gambiae* to *P. falciparum* relied either on genome-wide QTL mapping strategies [37], [38], [40] or on genotype-phenotype associations using a very limited number of candidate genes [36]. Here, we conducted a large-scale candidate gene association study based on a set of 67 *A. gambiae* immune-related genes selected based on their role in innate immunity [10] or their functional implication in the response to *Plasmodium* [14]. We identified associations between gene polymorphisms and *P. falciparum* infection success in a semi-natural system consisting of a newly established mosquito colony experimentally exposed to field parasite isolates. We verified the validity of the approach by confirming the functional role of one of the genes found to be associated with infection phenotype.

## Results

### Experimental Infections

Three separate experimental infections were carried out, each using a different gametocyte-positive blood isolate from carriers in Cameroon, hereafter named Isolate 1, 2 and 3. The number of mosquitoes included in the analysis (that fed and survived until dissection 8 days after the infectious blood meal) for Isolates 1, 2 and 3 were 380, 340 and 201, respectively. The *P. falciparum* isolates contained 813, 31 and 107 gametocytes/µl and infected 75%, 63% and 71% of the mosquitoes, with a mean number of oocysts per midgut of 11.5, 5.3 and 15.2, for Isolates 1, 2 and 3 respectively. Thus, around one third of all mosquitoes remained uninfected after feeding on the same infectious blood.

### Parasite Genotyping

To evaluate *P. falciparum* genetic diversity in the blood isolates used for experimental infections, two parasite merozoite surface protein (MSP) alleles were genotyped. This analysis revealed that the three isolates used in this study contained distinct *P. falciparum* genotypes. *MSP1 M* had three alleles in Isolate 1 and one in Isolates 2 and 3, and none were shared (one null allele was found). One, four and two *MSP2 FC* alleles were identified in Isolates 1, 2 and 3, respectively, with Isolates 1 and 3 sharing one allele. Although the MSP alleles identified in the blood samples did not necessarily represent the gametocyte population (the sexual stage infectious to the mosquito) at the time of infection, the allelic pattern displayed by each parasite isolate indicated that they were genetically distinct. This confirmed that *P. falciparum* populations in sub-Saharan Africa are genetically diverse and that each human infection generally consists of multiple parasite strains during the period of highest transmission [41]–[44].

### Mosquito Genotyping

157 single nucleotide polymorphisms (SNPs) located within and upstream 67 immune-related genes were successfully genotyped for mosquitoes fed on Isolate 1 with an extreme phenotype (see Methods). Based on their statistical significance, 21 of the 157 SNPs were selected for genotyping of mosquitoes with an intermediate phenotype and six of these were further selected for genotyping of mosquitoes fed on Isolates 2 and 3. Significant deviations from Hardy Weinberg Equilibrium (HWE) were detected in 18% of SNPs genotyped for mosquitoes with an extreme phenotype fed on Isolate 1 (data not shown), in 38% of SNPs when all mosquitoes fed on Isolate 1 were pooled (Table S1), and in 39% of the SNPs genotyped across all three isolates (Table S2). Because deviations from HWE affected a relatively large proportion of the SNPs, nine genes representing the range of Fis values were selected and sequenced to verify that the sequence matched the assigned genotype. Each gene contained between one and three of the selected SNPs with a total of 20 SNPs sequenced from the original DNA stock before whole genome amplification. Nucleotide identity ranged between 85 and 100% for each SNP with an average of 95%, which ruled out the possibility that the significant HWE deviations observed were due to technical errors. A possible biological explanation is that the mosquitoes used for each infection derived from a relatively small number of parental pairs, resulting in some degree of population structure in the offspring. Of ten neutral microsatellite loci that were genotyped, however, none were found to be in HW disequilibrium (Table S3), indicating that any genetic stratification must have been minor. It is worth mentioning that although HW disequilibrium may have affected the power of the study (i.e. the ability to detect genotype-phenotype associations), it did not affect the significance of the results (i.e. the probability of falsely rejecting the null hypothesis that a SNP is not associated with the phenotype).

### Linkage Disequilibrium

Overall, linkage disequilibrium (LD) between SNPs was low. A cut-off of r^2^ = 0.8 is commonly used to exclude redundant SNPs in association studies because a non-causative SNP in LD with a causative SNP will generally be found associated to the phenotype if r^2^≥0.8 [45]. Between 0.6 and 2.1% of SNP pairs were above this cut-off depending on the chromosome arm (Table 1). Of the 21 SNP pairs over this threshold, 67% were located less than 500 nucleotides apart, 29% were between 0.5 and 5.5 kb from each other and one SNP pair located 5.2 Mb apart showed long-range LD.

**Table 1:** Physical distance between SNP pairs in high linkage disequilibrium (r^2^≥0.8).

Chromosome	SNP 1	SNP 2	r^2^	Distance (bp)
3R	*CLIPB15-44101810*	*SCRBQ2-49293744*	0.83	5191934
2R	*SpSNAKElike-40688798*	*SpSNAKElike-40693950* *	0.88	5152
X	*CEC2-12437668*	*CEC3-12441793*	0.92	4125
3L	*CASPS1-35503929*	*CASPS4-35507474*	0.82	3545
2L	*APL2-18784235*	*APL2-18786047*	0.90	1812
3R	*CLIPB15-44100085*	*CLIPB15-44101810*	0.80	1725
2L	*CTLMA6-14231217*	*CTLMA2-14232746*	0.86	1529
3R	*CLIPB15-44099653*	*CLIPB15-44100085*	0.93	432
2R	*CLIPB17-7277304*	*CLIPB17-7277884*	0.84	409
2R	*AgMDL2-28317909*	*AgMDL2-28318283*	0.95	374
3L	*AgMDL1-40911948*	*AgMDL1-40912305*	0.90	357
2L	*ICHIT-31789378*	*ICHIT-31789719*	0.80	340
2R	*SpSNAKElike-40688481*	*SpSNAKElike-40688798*	0.82	317
2L	*APL2-18786108*	*APL2-18786414*	0.84	306
2L	*CASPS6-6045698*	*CASPS6-6045942*	1.00	244
2L	*APL1c-41258962*	*APL1c-41259161*	0.85	199
2R	*SpSNAKElike-40693407*	*SpSNAKElike-40693588*	1.00	181
3L	*TEP1-11202674*	*TEP1-11202849*	0.88	175
3R	*CLIPB15-44100085*	*CLIPB15-44100114*	0.86	29
2R	*APOD-23675873*	*APOD-23675896*	0.89	23
3L	*CTL2-8869856*	*CTL2-8869869*	0.96	13

*SNP associated to phenotype.

### Associations between SNPs and Infection Phenotype

When considering only the mosquitoes with an extreme phenotype fed on Isolate 1 (n = 192 females), significant association between the genotype and the level of infection was found in 21 of the 157 SNPs examined. After inclusion of intermediate phenotypes fed on the same isolate (n = 380 females), six SNPs remained significantly associated to infection phenotype. These SNPs are named as follows: *AgMDL1-40910564*, *CEC1-12441661*, *CLIPB4-34473971*, *SpPPOact-58805968*, *SpSNAKElike-40693950*, *TOLL6-41490803* (associated gene name-genomic position). The statistical significance of the associations was assessed through a False Discovery Rate (FDR) procedure to correct for multiple testing. If all null hypotheses (SNP genotypes are not associated to infection phenotype) were true, the FDR procedure would find zero significant tests in 95% of replicate studies and one significant test in 5% of replicate studies. The robustness of the six significant genotype-phenotype associations was evaluated across different parasite genotypes by repeating the experimental infections using three different genetically distinct *P. falciparum* isolates (Table 2). The parasite isolate used for infection generally had a significant effect on both components of the infection phenotype, prevalence (proportion of infected mosquitoes) and intensity (number of oocysts in infected mosquitoes). This isolate effect encompasses inherent experimental variation due to the day of infection as well as the genetic identity of the parasite isolate ingested. Overall tests across all isolates and both phenotypes confirmed genotype-phenotype associations showing significance for all SNPs with the exception of *CLIPB4-34473971*, which was marginally non-significant. Breaking down the analysis of the infection phenotype into tests of a total SNP effect within each component (prevalence and intensity), a SNP additive effect and an Isolate x SNP interaction effect (Table 2) provided additional information on the lack of significance of *CLIPB4-34473971*. Although none of the effects were significant, both interaction *P*-values were low, suggesting that the lack of overall effect may have resulted from the absence of genotype-phenotype association with Isolates 2 and 3.

**Table 2:** Statistical analysis of associations between SNP genotypes and infection phenotype including three *P. falciparum* isolates.

SNP Name	Factor	Prevalence *P*-value (χ2)	Intensity *P*-value (F test)
*AgMDL1-40910564*	SNP total effect	0.4401	0.0082**
	SNP additive effect	0.4452	0.0029**
	Isolate x SNP interaction	0.3755	0.2244
*CEC1-12441661*	SNP total effect	0.0017**	0.1399
	SNP additive effect	0.0002***	0.0941
	Isolate x SNP interaction	0.4546	0.2939
*CLIPB4-34473971*	SNP total effect	0.1500	0.0862
	SNP additive effect	0.7291	0.3015
	Isolate x SNP interaction	0.0659	0.0700
*SpPPOact-58805968*	SNP total effect	0.0031**	0.7154
	SNP additive effect	0.0010*	0.3606
	Isolate x SNP interaction	0.2486	0.8342
*SpSNAKElike-40693950*	SNP total effect	0.0739	<0.0001***
	SNP additive effect	NA	NA
	Isolate x SNP interaction	0.0320*	<0.0001***
*TOLL6-41490803*	SNP total effect	0.0082**	0.3159
	SNP additive effect	NA	0.4681
	Isolate x SNP interaction	0.0478*	0.2370

The table reports test statistics for the minimal models. NA means that no test of SNP additive effect was performed due to the significance of an Isolate x SNP interaction. A SNP total effect represents the overall effect of the SNP genotype on oocyst distribution (prevalence or intensity) across isolates. A SNP additive effect indicates that the SNP genotype effect has the same trend across isolates. An Isolate x SNP interaction shows that the SNP genotype effect has different trends across isolates. *P<0.05, **P<0.01, ***P<0.001.

**Figure 1:**
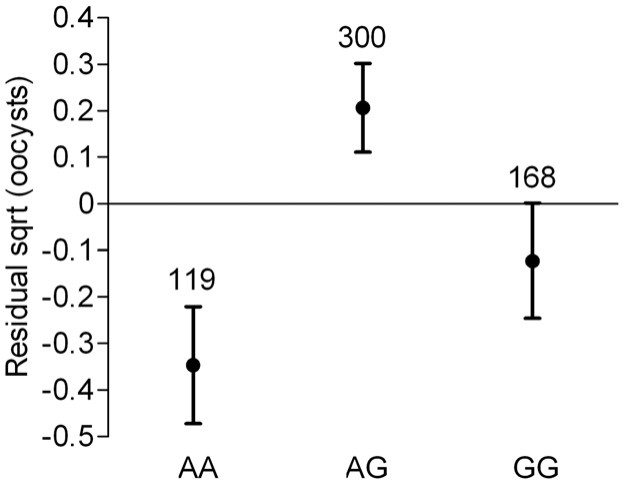
Association between *AgMDL1-40910564* genotype and *P. falciparum* infection intensity. The square root transformed mean number of oocysts in infected females and their standard errors are shown for each genotype. For clarity, plotted values are corrected for the additive isolate effect so that visual differences only reflect the genetic contribution to phenotypic variation (see Methods). Sample sizes are indicated above the bars.

*AgMDL1-40910564* genotype was associated to infection intensity, but not prevalence. The genotype-phenotype pattern was similar across the three parasite isolates as confirmed by the significant SNP additive effect and non-significant SNP x Isolate interaction (Table 2), suggesting that the effect of this SNP may be independent of parasite genotype. Heterozygotes were significantly more susceptible to *P. falciparum* infection than both homozygote genotypes (Figure 1). *AgMDL1-40910564* is located within the coding region of *AgMDL1*, which encodes a PRR [14]. The two alleles observed in the population (A and G) correspond to a synonymous substitution, suggesting that the causative SNP(s) are likely to be distinct. As mentioned previously, LD is generally low in the *A. gambiae* colony used in this study. The nearest known gene to *AgMDL1* is >1 kb away, making it likely that the causative SNP(s) are located within the same gene and lead to an amino acid modification altering parasite recognition. *AgMDL1* is thought to initiate an immune response upon recognition of a parasite, similar to its vertebrate homolog [14]. The gene is up-regulated 1.7 fold in response to *P. falciparum* infection and its silencing facilitates *P. falciparum* oocyst development but has little effect on *P. berghei*. Although prevalence and intensity are confounded in the analysis, *AgMDL1* appears to affect both components of the infection phenotype [14]. The identification of the association between *AgMDL1-40910564* with infection intensity in addition to the effect of *AgMDL1* silencing previously observed highlights the major role of this gene in controlling *P. falciparum* infection and the interest of deciphering its function. It is surprising that in the present study *AgMDL1-40910564* heterozygotes showed higher infection levels across three different isolates. One potential explanation is that when the parasites contained in the blood meal are genetically diverse (as in this study), different allelic forms of *AgMDL1* allow recognition of different *Plasmodium* genotypes, resulting in reduction of within-host competition between parasite strains and increased infection level in heterozygotes [46], [47]. The hypothesis of a parasite genotype-specific function of *AgMDL1* contrasts with the previous observation that the effect of *AgMDL1-40910564* did not depend on parasite isolate, but this interpretation may be complicated by complex interactions between co-infecting parasite strains [48] and requires further investigation.

**Figure 2:**
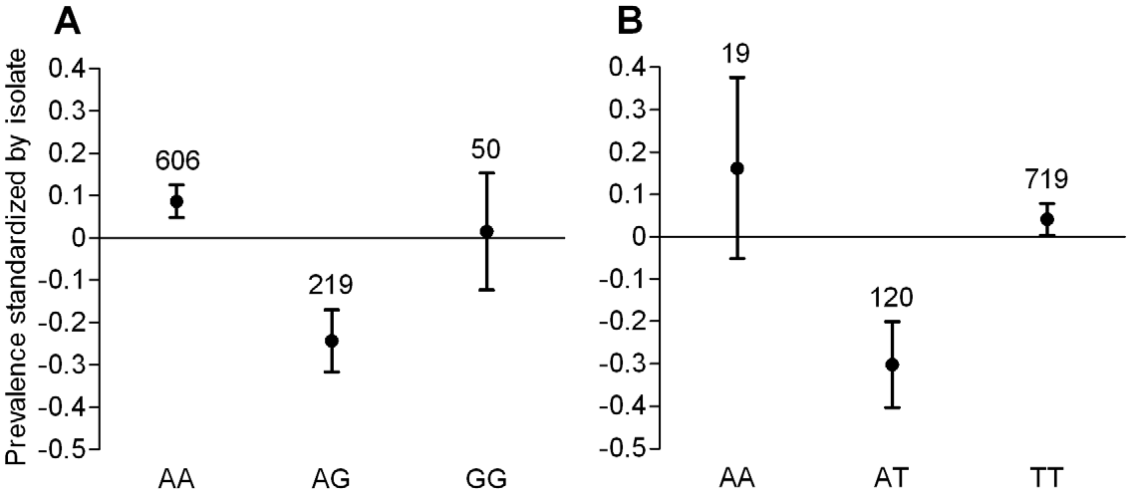
Association between *CEC1-12441661* and *SpPPOact-58805968* genotypes and *P. falciparum* infection prevalence. **A**) *CEC1-12441661* and **B**) *SpPPOact-58805968*. The proportion of infected females is shown for each genotype. For clarity, plotted values are corrected for the additive isolate effect so that visual differences only reflect the genetic contribution to phenotypic variation (see Methods). Vertical bars represent the confidence intervals of the standardized proportions. Sample sizes are indicated above the bars.

*CEC1-12441661* and *SpPPOact-58805968* genotypes were associated to infection prevalence, but not intensity. A similar pattern was observed across isolates as confirmed by the significant SNP additive effect, suggesting that the effect of these SNPs did not depend on parasite genotype (Table 2). Heterozygotes were significantly more resistant to *P. falciparum* infection than homozygotes (Figure 2). Higher resistance in heterozygotes is consistent with the idea that genetic heterozygosity enhances host immunity to infectious agents [49], [50]. *CEC1-12441661* is about 500 bp upstream from the coding region of *Cecropin 1* on one side and of *Cecropin 3* on the other side. Thus, this SNP could be causative or linked to causative SNP(s) in the regulatory regions affecting the expression of *Cecropin 1* or *Cecropin 3*, or linked to non-synonymous causative SNP(s) in the coding regions of either gene. Although *CEC1-12441661* was not in LD with any of the other SNPs genotyped on the chromosome, it is worth noting that two SNPs located on either side in *Cecropin 2* and *Cecropin 3* showed an r^2^ of 0.92 at a distance of 4.1 kb indicating that LD in this region can be high (Table 2). *Cecropin 1* and *Cecropin 3* are anti-microbial peptides for which different allelic variants could confer enhanced efficacy against a mixed-genotype *Plasmodium* infection. Consistently, allelic variants of *Cecropin 1* have previously been associated to natural *P. falciparum* infection [36]. *SpPPOact-58805968* is located 1.5 kb upstream of the coding region of *Sp PPO activate* but also in the coding region of the gene *AGAP004639* causing a synonymous mutation. This SNP could be causative by affecting regulation of *Sp PPO activate* expression or linked to causative non-synonymous SNP(s) in either of the genes. *Sp PPO activate* is part of a serine protease cascade up-regulated in response to *P. falciparum* infection [14] and thus represents a strong candidate gene whose polymorphism may underlie variation in resistance. *AGAP004639* is an ortholog of genes encoding clip domain serine proteases in *Aedes aegypti* and *Culex quinquefasciatus* involved in signal modulation and amplification following non-self recognition [10]. To our knowledge it has not already been implicated in the mosquito response against *Plasmodium* but is also a promising candidate. Causative SNPs within either gene will likely interact indirectly with the parasite altering the amount or type of effector molecule produced.

**Figure 3:**
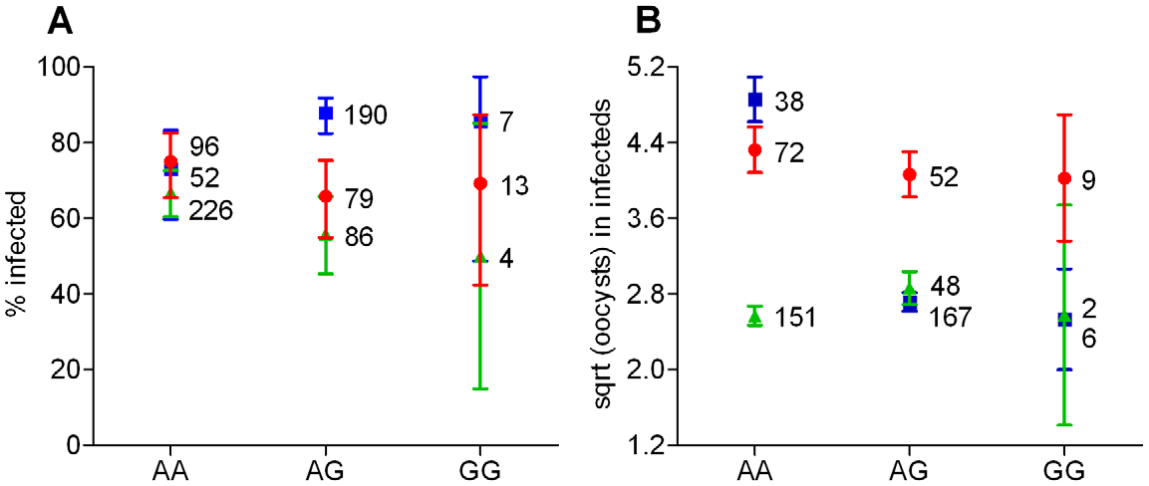
Association between *SpSNAKElike-40693950* genotype and infection phenotype as a function of *P. falciparum* isolates. **A**) Percentages of infected females (infection prevalence) and their confidence intervals. **B**) Numbers of oocysts in infected females (infection intensity) and their standard errors. Sample sizes are indicated next to each data point. Blue squares: Isolate 1; green triangles: Isolate 2; red circles: Isolate 3.

*SpSNAKElike-40693950* genotype was significantly associated to infection intensity and marginally to prevalence. In both cases, associations were parasite isolate-specific as indicated by the significant Isolate x SNP interaction (Table 2). For infection prevalence, AA homozygotes showed greater resistance than AG heterozygotes against Isolate 1, but the opposite trend was observed with Isolates 2 and 3 (Figure 3A). For intensity, AA homozygotes showed greater susceptibility than AG heterozygotes against Isolate 1, and to a lesser extent Isolate 2. The opposite trend was observed with Isolate 3, although the effect was relatively modest (Figure 3B). The non-significant total SNP effect on prevalence indicates that this association was weaker than for intensity (Table 2). The significant Isolate x SNP interactions suggest that the effect of this SNP, or linked causative SNP(s), on the outcome of infection depends on the parasite genotype. Although the SNP effect is highly significant for Isolate 1, it is not significant with either Isolates 2 or 3 alone, as shown by separate Kruskal-Wallis tests for each isolate (data not shown). The strong association between *SpSNAKElike-40693950* genotype and infection phenotype for Isolate 1 but not for the two other isolates is consistent with the potential implication of this SNP, or linked causative SNP(s), in specific G x G interactions with the parasite. The SNP is located in the coding region of *Sp SNAKElike,* which is involved in a serine protease cascade and is up-regulated following *P. falciparum* infection [14]. It causes a synonymous mutation and is therefore likely to be linked to causative SNP(s). Although the nearest gene is>1.2 kb away, this particular SNP is in high LD with another SNP located 5.2 kb away, pointing to a larger region that may contain the causative SNP(s). The causative SNP may act indirectly with the parasite affecting the downstream immune signal.

**Figure 4:**
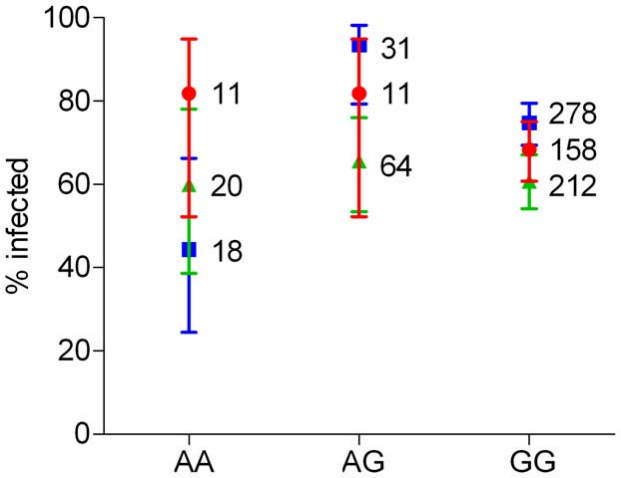
Association between *TOLL6-41490803* genotype and infection prevalence as a function of *P. falciparum* isolates. The percentages of infected females and their confidence intervals are shown by isolate for each genotype. Sample sizes are indicated next to each data point. Blue squares: Isolate 1; green triangles: Isolate 2; red circles: Isolate 3.

Isolate-specific association was also observed between *TOLL6-41490803* genotype and infection prevalence, as indicated by the significant Isolate x SNP interaction (Table 2). For Isolate 1, heterozygotes were more susceptible to infection than both homozygote genotypes (Figure 4), a similar scenario to *AgMDL1-40910564*. Isolates 2 and 3 showed a similar trend, although the effects were not statistically significant when analyzed independently by a Kruskal-Wallis test (data not shown). The significant Isolate x SNP interaction suggests that this SNP, or linked causative SNP(s), may be responsible for G x G interactions with the parasite. The SNP is located within *TOLL6*, which encodes a toll-like receptor involved in immune signal transduction [10], [51] causing a non-synonymous mutation. This could therefore be causative or be linked to causative SNP(s) probably located within the same gene (the nearest known gene is>0.5 Mb away). This SNP may act indirectly with the parasite affecting the downstream immune signal.

### Functional Role of *Sp SNAKElike* in *P. falciparum* Development

**Figure 5:**
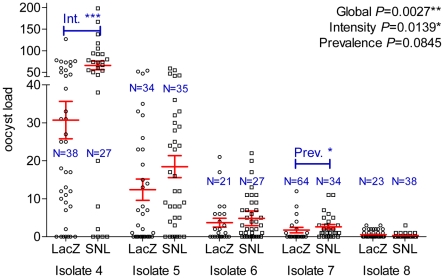
Effect of *A. gambiae Sp SNAKElike* (SNL) silencing on *P. falciparum* infection. The number of oocysts that developed for mosquitoes injected either with ds*Sp SNAKElike* or ds*LacZ* in five independent gene knockdown assays using different *P. falciparum* isolates are shown. The red bars indicate the mean numbers of oocysts and their standard error. Sample sizes are indicated. *P<0.05; **P<0.01; ***P<0.001.

Statistical association between a genetic marker and infection phenotype does not provide conclusive evidence that the gene where the genetic marker is located plays a functional role in controlling infection. We verified the validity of our approach by testing the functional role of *Sp SNAKElike* in *P. falciparum* infection through gene silencing assays using five new *P. falciparum* isolates (named 4–8 hereafter). *Sp SNAKElike* was selected due to the level of significance of *SpSNAKElike-40693950* to both components of infection phenotype (intensity and prevalence), together with the longer range LD exerted by this SNP decreasing the specificity of the association. Overall, RNAi knockdown of *Sp SNAKElike* resulted in increased susceptibility to *P. falciparum* infection (Figure 5). In a combined analysis of prevalence and intensity, mosquitoes depleted for *Sp SNAKElike* expression harbored significantly more oocysts per midgut than control mosquitoes (overall *P*-value = 0.0027). Further analysis showed that *Sp SNAKElike* silencing had a main effect on infection intensity (*P* = 0.01) but not prevalence (*P* = 0.08), which was consistent with the results of the association study. The amplitude of the effect varied with the isolate. As *SpSNAKElike-40693950* is in high LD with another SNP 5.2 kb away, the region potentially containing the causative SNP(s) in the association study is relatively large. Increased oocyst numbers upon *Sp SNAKElike* knockdown confirm that this gene is an antagonist to *P. falciparum* development and makes it likely that the causative SNP(s) include *SpSNAKElike-40693950* and/or closely linked SNP(s) within the same gene. This gene was selected for inclusion in the study based on its up-regulation in response to ingestion of a *P. falciparum* infected blood meal [14]. *Sp SNAKElike* in *Drosophila* is up-regulated in response to Gram-positive bacteria and fungi and predicted to activate the TOLL immune response pathway [52]. It is therefore likely that *Sp SNAKElike* plays a role in immune signaling in *A. gambiae*.

## Discussion

This study is, to our knowledge, the first one that examined associations between natural polymorphisms in a large number of immune-related genes in *A. gambiae* and *P. falciparum* infection success. Out of 67 initial candidates, five immune genes had polymorphisms that strongly associated with infection phenotype through our screening scheme. Genetic variation in these genes (or closely linked loci) thus contributes to phenotypic variability of *A. gambiae* resistance to *P. falciparum* in natural populations. Although our approach bears some limitations (discussed below), it provides several new insights into our understanding of the genetic basis of *A. gambiae* resistance to *P. falciparum* in natural populations. Our results show that genes underlying variation in infection intensity may be partly distinct from those controlling variation in infection prevalence. Importantly, some of the associations found were isolate-specific, indicating that the alleles underlying *A. gambiae* resistance or susceptibility to *P. falciparum* can differ across parasite genotypes. We validated our approach by confirming the functional role of a gene that contained a SNP significantly associated to infection phenotype.

Earlier studies have successfully used a genome-wide QTL mapping strategy in field-derived isofemale mosquito families to detect associations between microsatellite markers and *P. falciparum* infection phenotype [37], [38], [40]. Here we tested associations between SNPs identified in a large set of candidate immune genes and infection phenotype in a newly established mosquito colony. Although our method likely excluded potentially important genes because of the initial selection of candidates, it has the major advantage of the minimal LD harbored by a randomly mating colony compared to isofemale pedigrees for which LD spans over genomic regions of several Mb. Whereas associations found using isofemale pedigrees necessitate to gradually refine genetic mapping to identify the causative polymorphisms, our strategy likely identified SNPs that are either causative or closely linked to the causative SNPs [53]. The *Plasmodium* Resistance Island 1 (PRI1) previously uncovered in natural populations of *A. gambiae* [37] includes several immunity genes but the causative polymorphic sites remain to be identified. In the present study, 12 SNPs were genotyped in the PRI1, within and upstream of *APL1* and *APL2*, but none were associated to infection phenotype. This emphasizes the need for complementary approaches to unravel the complex genetic basis of mosquito resistance to *P. falciparum* in natural populations.

Polymorphisms in the five genes identified in the present study most likely act in concert with genetic variation in many other genes to drive phenotypic variability [54]. In other words, the five candidate genes that we identified probably represent only a glimpse into the complex genetic basis of *A. gambiae* resistance to *P. falciparum* in natural populations. Several aspects of our approach that were designed to increase our power of detection also limited its scope. Firstly, our study was initiated with a set of candidate genes known to be implicated in the mosquito immune response. Most of these genes have been identified in functional studies based on their up- or down-regulation upon *Plasmodium* infection [14]. That a gene is functionally involved in anti-*Plasmodium* immunity does not necessarily imply that its polymorphism underlies phenotypic variation in resistance. Conversely, other genes that are not regulated upon infection may contribute to phenotypic variation in resistance. Thus, by using a selected set of genes based on the available data we inevitably excluded an unknown number of potential candidates. Secondly, the first two steps of our procedure excluded SNPs that did not show a significant association to infection intensity by a single *P. falciparum* isolate. As a result, genetic polymorphisms acting on prevalence and/or involved in isolate-specific resistance may have been missed. Ideally, equal numbers of mosquitoes from different infections should be genotyped before selecting SNPs for more stringent analyses. Such a strategy would be less likely to exclude SNPs only effective against a subset of isolates.

By decomposing the infection phenotype into infection prevalence and infection intensity, we found that four of the five SNPs significantly associated to infection phenotype were associated to only one but not both components. This indicates that different mechanisms, involving different gene pathways, may control the different steps of the infection process. We hypothesize that the pathways triggered to prevent infection differ to some extent from those involved in minimizing infection intensity. Differences in the genetic basis underlying *Plasmodium* infection prevalence and infection intensity in *Anopheles* mosquitoes have been previously observed [39], [55]. For two of the five significant SNPs (*AgMDL1-40910564* and *TOLL6-41490803*) heterozygous mosquitoes had an increased parasite load, which was unexpected as heterozygosity is generally expected to increase resistance [49], [50]. When previously observed [56]–[58], it has been hypothesized that both alleles alone may have beneficial effects in homozygotes if reduced protein production of each of the two allelic variants in heterozygotes leads to reduced fitness [59]. Both under-dominance (homozygote advantage) and over-dominance (heterozygote advantage) are suggested in the associated SNPs, both of which were shown to have important consequences for the maintenance of polymorphism in immunity genes [60]–[62]. Our results suggest that evolutionary forces maintain polymorphism in the *A. gambiae* immune system, although so far purifying selection was identified as the most common form of selection [63]–[66].

By explicitly accounting for parasite variation in the analysis, we identified SNPs that were associated with infection phenotype in an isolate-specific manner. Two of the five significant SNPs (*SpSNAKElike-40693950* and *TOLL6-41490803*) had an effect that strongly depended on the parasite isolate. By replacing the autologous serum with naïve serum, we standardized the infectious blood meal that was offered to the mosquitoes. We did not control, however, potential differences in the nutritive quality of the blood meal, which may have affected parasite infection success. Nonetheless, isolate-specific associations detected between SNPs and infection phenotype are consistent with earlier evidence that *P. falciparum* infection in *A. gambiae* is governed by G x G interactions [26], [67]. The present findings go one step further by providing evidence for specific mosquito genotype by parasite isolate interactions (an approximation of G x G interactions) at the molecular level. That only two SNPs showed significant SNP x Isolate interactions indicates that among *A. gambiae* genes underlying resistance/susceptibility to *P. falciparum*, only some may be responsible for G x G interactions whereas others would provide a generalist effect against all parasite genotypes. For example, it was observed that *APL1A* had a similar effect across multiple *P. falciparum* genotypes [39], [55]. By measuring significant SNP x Isolate interactions for *SpSNAKElike-40693950* and *TOLL6-41490803*, we may have found the two first genes governing specific compatibility between *A. gambiae* and *P. falciparum*.

The suggestion of mosquito genotype by parasite isolate interactions at the SNP level has important implications for current efforts towards identification of *Anopheles* genes underlying resistance to *P. falciparum* [67]. Genetic dissection of mosquito resistance to *Plasmodium* is usually conducted either in model systems or in semi-natural systems that do not account for the genetic variation among parasites (e.g. [6], [11], [14], [37], [39], [40], [68]). Data obtained from infections using different parasite isolates are generally pooled or the effect of genetic polymorphism determined by computing statistical significance across experiments. The present study reveals the importance of considering the variation observed between infections with different parasite isolates in functional genetic or association studies. A significant portion of the observed variation in infection phenotype seems to result from specific interactions between mosquito and parasite genotypes. A total SNP effect was found for two of the three SNPs involved in significant Isolate x SNP interaction effects, but when each of the three isolates was considered separately the SNP effect was only significant for Isolate 1. Previous studies may therefore have reported significant genotype-phenotype associations based on a total effect and presumed to be parasite isolate-independent that may have in fact resulted from an interaction effect. This highlights the need to integrate the effect of the parasite genome in interaction with the mosquito genome if we are to fully understand the genetic architecture of mosquito resistance to malaria parasites [69].

LD reduces the specificity of associations and is highly influenced by chromosomal inversions [70]. The mosquito strain used in the current study is fixed for the Forest (standard) pattern of inversions [71], so that genomic regions with significantly increased LD due to chromosomal inversions are not expected. In future studies using mosquito strains with polymorphic inversion patterns, specific SNP locations and LD relationships will have to be carefully examined to interpret the results.

Detecting associations between infection phenotype and individual SNPs supports the potential functional importance of the genes in which they lie, whether the association is isolate-specific or not. Most of the genes in which we uncovered significant SNPs have been relatively well characterized for their role in the immune response of *A. gambiae* or closely linked species [10], [14], [36], [51]. The exact role of TOLL6 is still unknown, but it may play a role in the TOLL pathway, which has been mainly implicated in the *A. gambiae* response to *P. berghei* and not *P. falciparum* [39]. A recent study, however, suggests a role for the two major immune signaling pathways, TOLL and IMD, with the IMD pathway controlling infection prevalence and the TOLL pathway controlling infection intensity [19]. The present study supports a role for both pathways although *TOLL6-41490803* showed association to infection prevalence, suggesting that some aspects of these major immune pathways in the mosquito remain to be discovered. Little is known, however, about the functional role of *Sp SNAKElike,* which contains the SNP with the most significant association in this study, besides its up-regulation in *A. gambiae* in response to ingestion of a *P. falciparum*-infected blood meal [14]. We used RNAi gene knockdown to show that *Sp SNAKElike* plays an important role in controlling infection with a major effect on infection intensity. This result is consistent with the hypothesis that ‘*SNAKE* like’ genes are responsible for activating the TOLL immune pathway in *Drosophila* [52], which in *A. gambiae* plays a major role in controlling *P. falciparum* infection intensity [19]. The phenotypic effect of *Sp SNAKElike* silencing supports the conclusion that the causative SNP(s) in the association study are located within this gene rather than further away, as could have been suggested by the LD data. The variation observed in the gene knockdown effect across isolates could result from mosquito genotype by parasite isolate interactions in agreement with the association analysis. Such isolate-dependent variation in the functional effect of gene silencing is expected if the function of the gene depends on a specific interaction between its own polymorphism and that of one or several parasite genes. In particular, when the gene variant(s) present in a mosquito genotype does not allow effective recognition or interaction with a certain parasite genotype, gene silencing will lead to little or no phenotypic difference for this isolate.

To conclude, we used a multi-step association procedure that provided strong support for the role of genetic variation within or near five candidate immune genes in natural resistance of *A. gambiae* to *P. falciparum*. The relevance of our approach was validated functionally for the candidate genes identified in the association analysis. Although our approach was not exhaustive, this information will be useful for future allele-specific functional characterization of the corresponding genes or their immediate genomic region. In addition, our association analysis at the SNP level provided important information for further dissection of the genetic basis of natural *A. gambiae* resistance to *P. falciparum*. Four of the five significant SNPs were associated to either the probability of infection or the parasite load, but not both, indicating that genetic variation underlying infection prevalence likely differs from that underlying infection intensity. The effect of two SNPs on infection phenotype was isolate-specific, suggesting that G x G interactions in this system likely occur at the gene level. This information will be useful to identify molecular targets for strategies aimed at interrupting malaria transmission during parasite development in the vector.

## Methods

### Ethical Statement

Ethical approval was obtained from the Cameroonian National Ethics Committee. All human volunteers were enrolled after written informed consent from the participant and/or their legal guardians.

### Study Area

Mosquitoes and blood donors came from the vicinity of Yaoundé, a rainforest area in Cameroon, where the intensity of malaria transmission is relatively constant throughout the year, but slightly higher during the rainy seasons [72].

### Mosquito Colony

An *A. gambiae* s.s. colony was established in January 2006 from larvae collected in Ngousso, a suburb of Yaoundé and reared at the OCEAC insectary under standard conditions (12 h day/night cycle, 28 +/− 2°C, 85 +/− 5% humidity, adults maintained on 8% sucrose). The mosquito colony, named “Ngousso”, was used within 6 months from its establishment to limit the loss of polymorphism due to maintenance under laboratory conditions. The colony is of the M molecular form and Forest chromosomal form.

Potential genetic structure in the mosquito colony was tested to confirm that the adult mosquitoes were freely interbreeding. Ten neutral microsatellite markers distributed throughout the genome were genotyped for 100 randomly selected mosquitoes from the colony and potential deviations from HWE measured in Genepop [73] and corrected for multiple testing using the Bonferroni procedure. The loci used were AG3H119, AG3H242, AG3H249, AG3H555, AG3H577, AG3H59, AG3H746, AG3H812, AG3H817 and AG3H93 [74].

### Experimental Infections

*P. falciparum* gametocyte carriers were selected by examining thick blood smears from school children aged between five and eleven, who lived and attended school in Mfou, a small town located 30 km from Yaoundé. Malaria positive individuals were treated according to national recommendations. Up to 8 ml of venous blood was taken from selected carriers with at least 20 gametocytes/µl of blood (estimated based on an average of 8000 white blood cells/µl). In order to limit the potential effect of human transmission blocking immunity [75], the blood was first centrifuged at 2000 rpm at 37°C for three minutes and the serum changed to European naive AB serum with 0.225 UI heparin/ml (to prevent clotting). 500 µl of reconstituted blood was added to membrane feeders maintained at 37°C by water jackets. Two to three day-old female mosquitoes were allowed to feed for up to 30 minutes through a Parafilm membrane. Un/partially fed mosquitoes were removed and fully fed mosquitoes maintained under standard conditions on an 8% sucrose diet. At day eight post infection, midguts were dissected in 0.4% mercurochrome stain and the infection load of each individual female was determined by counting oocysts under a light microscope and carcasses kept for genotyping. This procedure was repeated three times, each experimental infection using a different gametocyte carrier, referred to as Isolate 1, 2 and 3.

### Parasite Genotyping

DNA was extracted from an aliquot of the blood used in each infection using DNAzol (Medical Research Centre) and parasites were genotyped at the *MSP1 M* and *MSP2 FC* loci by measuring nested PCR fragment lengths as previously described [76]. Here, we used fluorescently labeled reverse primers and detected sizes on an Applied Biosystems 3130xl Sequencer.

### Mosquito SNP Identification and Genotyping

Names of the 67 genes included in this study are either from VectorBase (http://www.vectorbase.org/) or published literature (see Table S4 for corresponding VectorBase gene IDs). Genes were selected to represent the range of immune families previously characterized [10] with emphasis put on those implicated in the *A. gambiae* response to *Plasmodium* (e.g. [14]) and were amplified using previously published [37], [63], [64] and newly designed primers. Sequences were obtained from VectorBase and primers designed with Primer 3 [77] (Table S4) to amplify approximately 700 bp upstream and/or within coding regions of each selected gene. These regions were amplified by PCR using 25 µl reaction mixes as previously described [63] for eight mosquitoes from the Ngousso colony. PCR products were sequenced using the Big Dye Terminator v3.1 Sequencing Kit (Applied Biosystems), and run on an Applied Biosystems 3130xl Sequencer. Sequences were verified using SeqScape (Applied Biosystems) and aligned in Mega v3.1 [78]. 157 SNPs found more than once in the eight mosquitoes were selected. Genotyping single base pair extension primers were designed directly upstream of each SNP using Oligo Explorer and Oligo Analyser (http://www.cmbn.no/tonjum/biotools-free-software.html). GACT repeat tails were added to genotyping primers to allow pooling of up to ten per reaction, and migration distances tested using the SNaPshot Primer Focus kit (Applied Biosystems).

For genotyping, DNA was isolated from the remaining mosquito carcasses after midgut dissection as previously described [79] and the Genomiphi kit applied (Amersham) for whole genome amplification in an unbiased manner [80]. The SNaPshot method (Applied Biosystems) was used to genotype mosquitoes before samples were run on an Applied Biosystems 3130xl Sequencer and results analysed using GeneMapper v4.1 software (Applied Biosystems). For each genotyped SNP, deviations from HWE were determined as described above.

### Statistical Analysis

The procedure consisted of three steps. Firstly, 192 females fed on Isolate 1 with an extreme phenotype [81], of which 96 had 0–1 oocysts/midgut and 96 had 14+ oocysts/midgut, were genotyped for all 157 SNPs. The Kruskal-Wallis test was applied in R v.2.10.0 [82] to detect significant genotype-phenotype associations. Specifically, this test compares oocyst counts between the three possible genotypic categories (heterozygotes and both types of homozygote). Secondly, significant SNPs from the first step of the analysis were genotyped in all of the remaining females (n = 185) fed on Isolate 1 that had intermediate phenotypes (1–14 oocysts/midgut). Genotyping data from all individuals fed on Isolate 1 were combined into a full data set for the significant SNPs. These were reanalyzed using the Kruskal-Wallis test. An FDR analysis [83], [84] was used on the final *P*-values of per-SNP Kruskal-Wallis tests, with the original FDR procedure [85] applied at the 5% level. We therefore increased the sample size for the SNPs that were most notable in the 192 initial individuals before applying the FDR analysis. Although this procedure increases the detection power for SNPs with strong effects, it is conservative because it tends to decrease significance when there is no true genotype-phenotype association. The basic genotype-phenotype analysis for SNP filtering required up to this point was based on univariate, non-parametric tests. Thirdly, significant SNPs following the first two steps of the analysis (based on genotype-phenotype associations from a single *P. falciparum* isolate) were genotyped for all mosquitoes fed on Isolates 2 and 3, giving a data set including these SNPs across all three parasite isolates. Infection phenotype was decomposed into prevalence (proportion of mosquitoes with at least one oocyst) and intensity (number of oocysts in individuals with at least one oocyst). Prevalence was analyzed by binomial logistic models and intensity in infected individuals by linear models. In the latter analysis, oocyst numbers were square-root transformed to achieve normality of the residuals. For each individual SNP, both components of the infection phenotype were analyzed as a function of the mosquito genotype, the parasite isolate, and their interaction. Effects were tested by standard analysis of variance. To avoid multiple testing issues, each SNP was analyzed in a stepwise manner, starting with an overall test by Fisher's combination of probabilities method [86], combining the *P*-values of the intensity and prevalence analyses, and further analyzing significant results in terms of their components (prevalence or intensity). Likewise, in each case, the complete model (SNP and Isolate effects with interaction) was compared to the model with Isolate effect alone to obtain a single test of the total SNP effect (resulting from both additive and interaction effects). A total SNP effect means that the oocyst distribution (prevalence and/or intensity) differs depending on the SNP genotype. The total SNP effect was further analyzed by stepwise deletion of effects. When an Isolate x SNP interaction was statistically significant (*P*<0.05), the model with interaction was retained. An interaction effect means that the SNP effect on the oocyst distribution (prevalence and/or intensity) differs depending on the parasite isolate. When no significant interaction was detected, a SNP additive effect, i.e. measuring the same trend across isolates, was tested in a model with Isolate effect. All analyses were performed with the functions lm, glm and anova in the R software [82]. For graphical representation, when the SNP x Isolate interaction was not statistically significant, phenotypic values were corrected for the main effect of the isolate so that visual differences could be directly attributed to the genotypes. For infection prevalence the proportion was standardized by isolate, whereas for infection intensity we plotted the residuals of a one-way analysis of oocyst numbers as a function of isolate. When the SNP x Isolate interaction was statistically significant, no correction was made and the raw data was plotted separately for each isolate within the same graph.

### Linkage Disequilibrium

LD between SNP pairs was measured for each chromosome arm as r^2^ in Haploview [87]. A cut-off of r^2^ = 0.8 [45] was used to identify SNP pairs in high LD and estimate the accuracy, in terms of genetic distance, of the associations found.

### Gene Knockdown Assays

The gene *Sp SNAKElike* was functionally tested for its role against *P. falciparum*. Double stranded RNA (dsRNA) was produced using the T7 Megascript Kit (Ambion) as described previously [7], [68]. cDNA was obtained using *Sp SNAKElike* primers (SNL Inner F: 5′-TTGCACTGGTGAAGCTCAAG-3′; SNL Inner R: 5′-CCTTCGTGTAGATCGCCTTC-3′) with *A. gambiae* Ngousso RNA as a template. Double stranded *LacZ* RNA was used as a control. For both genes, *Sp SNAKElike* and *LacZ*, 200 ng of dsRNA was injected into the thorax of 1-2 day-old mosquitoes anesthetized with CO_2_, using a nano-injector (Nanoject II, Drummond Scientific) [7]. Up to 100 ds*LacZ* and ds*Sp SNAKElike* injected mosquitoes were experimentally challenged with an infectious blood meal four days post injection as described above and oocysts counted eight days later. A total of ten feedings with *P. falciparum* gametocyte positive blood isolates were performed. Only experiments with at least 20 live mosquitoes eight days after feeding in each treatment were included in the analysis.

Data from the gene knockdown assays were analyzed with non-parametric tests because no transformation of oocyst counts yielded normally-distributed residuals after linear modeling. Infection phenotype was analyzed in two steps. Firstly, total oocyst counts were compared with separate Wilcoxon Mann-Whitney tests for each isolate and *P*-values were combined using Fisher's meta-analysis approach [86]. Secondly, infection phenotype was decomposed into prevalence and intensity (as described above), which were compared for each isolate with an exact chi-square test for contingency tables and a Wilcoxon Mann-Whitney test, respectively. *P*-values were combined for each isolate using Fisher's meta-analysis approach. All performed in R [82].

Gene knockdown success was confirmed by semi-quantitative PCR from mosquitoes collected four days after dsRNA injection and prior to feeding. Total RNA was extracted from 15 mosquitoes using Trizol reagent (Invitrogen) and cDNA was synthesized using the SuperScript III Reverse Transciptase Kit and an oligo (dT) 20 primer (Invitrogen). The *A. gambiae S7* ribosomal gene was used to normalize the amount of RNA between knockdown and control mosquitoes. Semi-quantitative PCRs were conducted using the primers SNL Outer F 5′-ACGCTAATACCGCTCACGAT-3′ and SNL Outer R 5′-CCCCACACGTTGTCCTCTAT-3′ for *Sp SNAKElike* and S7 F 5′-AGGCGATCATCATCTACGTGC-3′ and S7 R 5′-GTAGCTGCTGCAAACTTCGG-3′ for *S7*.

## Supporting Information

Table S1 Kruskal-Wallis test for association and deviations from HWE. Data given for 21 SNPs (previously significant based on mosquitoes with extreme phenotype only) for all mosquitoes fed on Isolate 1. * *P*<0.05.
(0.02 MB XLS)

Table S2 Deviations from HWE for the final 6 SNPs for Isolates 1–3. * *P*<0.05.
(0.02 MB XLS)

Table S3 Deviations from HWE for 10 neutral microsatellite markers.
(0.02 MB XLS)

Table S4 Immune related gene, primer and SNP details. + indicates primers from [37], # indicates primers from [63] and ∧ indicates primers from [64] (Nested Primers).
(0.05 MB XLS)
